# Adult Gonococcal Conjunctivitis Complicated by Peripheral Ulcerative Keratitis: A Case Report

**DOI:** 10.1002/ccr3.72947

**Published:** 2026-06-14

**Authors:** Prashim Sharma, Ramesh Bikram Khatri Chhetri, Alisha Adhikari, Badri Prasad Badhu, Prerna Arjyal Kafle

**Affiliations:** ^1^ Birat Medical College Teaching Hospital Morang Nepal; ^2^ Department of Ophthalmology Birat Medical College Teaching Hospital Morang Nepal

**Keywords:** amniotic membrane grafting, case report, gonococcal conjunctivitis, *Neisseria gonorrhoeae*, peripheral ulcerative keratitis

## Abstract

*Neisseria gonorrhoeae* conjunctivitis is rare in adults but may rapidly progress to vision‐threatening complications. Peripheral ulcerative keratitis (PUK) is an unusual and severe manifestation. A 42‐year‐old male presented with bilateral ocular pain, swelling, and profuse purulent discharge for 15 days, leading to inability to open his eyes. History revealed recent urethral discharge and multiple sexual contacts. Ocular examination showed severe conjunctival chemosis and peripheral corneal thinning with descemetocele formation. Gram stain of conjunctival swabs demonstrated intracellular gram‐negative diplococci. Serology revealed positive VDRL and TPHA (late latent syphilis). HIV and *Chlamydia trachomatis* testing were unavailable. The patient received intravenous ceftriaxone 1 g twice daily, oral doxycycline, and topical fortified antibiotics. Due to progressive corneal thinning, bilateral amniotic membrane grafting with perilimbal conjunctival excision and bandage contact lens placement was performed. Syphilis was treated with three weekly doses of benzathine penicillin G. At 6 weeks, vision improved to 6/9 bilaterally with healed ulcers and clear corneas. Gonococcal conjunctivitis in adults can rapidly progress to peripheral ulcerative keratitis. Early diagnosis, broad STI screening, systemic therapy, and timely surgical intervention are critical for preserving vision.

## Key Clinical Message

Ocular gonorrhea in adults may cause rapid corneal destruction and peripheral ulcerative keratitis. Comprehensive STI evaluation and timely surgical management, including amniotic membrane grafting, are crucial to prevent irreversible blindness.

## 1 Introduction

*Neisseria gonorrhoeae* is a common sexually transmitted pathogen, but ocular involvement in adults is rare. When it occurs, conjunctivitis can progress rapidly to corneal ulceration and even perforation if untreated [1, 2]. Unlike neonatal disease, adult cases are infrequently reported and may present with severe complications. Peripheral ulcerative keratitis (PUK) secondary to gonococcal conjunctivitis is particularly uncommon, with only a handful of cases documented [3, 4]. Early recognition, systemic antibiotic therapy, and timely surgical intervention are critical to prevent irreversible vision loss. We report a rare case of adult gonococcal conjunctivitis complicated by bilateral PUK requiring amniotic membrane grafting.

## 2 Case History and Examination

A 42‐year‐old man from eastern Nepal presented with a 15‐day history of bilateral eye redness, foreign body sensation, and purulent discharge, progressing to marked swelling and inability to open his eyes. He also reported blurring of vision and severe discomfort.

He had experienced a self‐resolving episode of whitish urethral discharge 1 month earlier, associated with dysuria. He admitted to multiple unprotected sexual contacts in the preceding 2 months. He consumed alcohol frequently and smoked one pack every 2 days. There was no past history of trauma, ocular disease, or systemic illness.

### 2.1 Examination Findings

**FIGURE 1:**
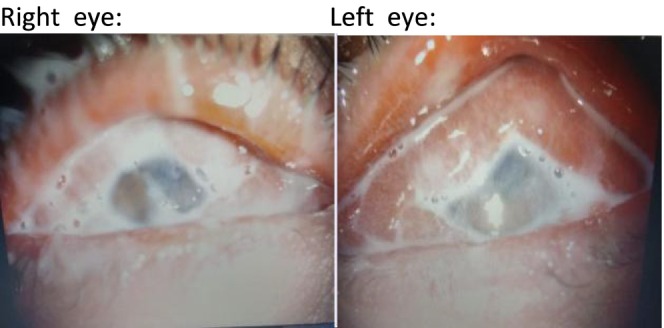
Both eyes showing congestion, chemosis, and purulent discharge on the first day of his visit.

- Vitals: stable (T 98.6°F, BP 110/90 mmHg, HR 82/min).
- General exam: normal.
- Ocular exam: bilateral chemosis, conjunctival congestion, and copious mucopurulent discharge (Figure 1). Visual acuity was 6/36 (right eye) and 6/60 (left eye). Peripheral corneal thinning with early descemetocele was noted.
- Genital exam: whitish urethral discharge at meatus. No skin lesions, ulcers, or lymphadenopathy.

## 3 Investigations and Treatment

### 3.1 Investigations

- Gram stain of conjunctival swabs: numerous polymorphonuclear cells with intracellular gram‐negative diplococci, consistent with *Neisseria gonorrhoeae* .
- Urethral swab: pus cells, no organisms seen.
- VDRL: reactive at 1:6 titre; TPHA: positive.
- HIV testing not available
- NAAT/culture for gonococcus and *Chlamydia trachomatis* not available (limitation)
- Routine blood tests: within normal range

### 3.2 Management and Course

**FIGURE 2:**
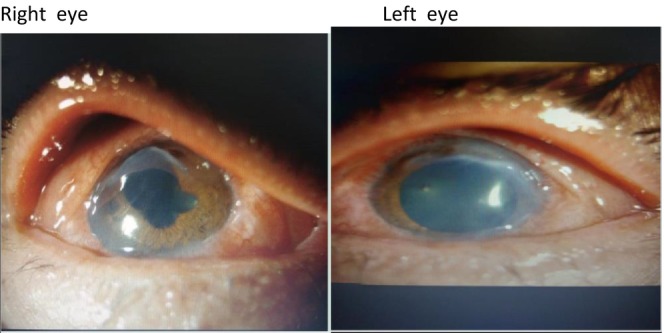
After 6 days of antibiotic use, corneal thinning was observed at the 11–1 o'clock positions in both eyes, as well as at the 9 o'clock position on the right side and the 3 o'clock position on the left.

- Started on intravenous ceftriaxone 1 g twice daily, oral doxycycline 100 mg twice daily for 7 days, topical fortified cefazolin and tobramycin, moxifloxacin drops, and lubricants.
- On day 2, indomethacin and vitamin C were added.
- After 6 days of antibiotics therapy, progressive corneal thinning with bilateral descemetocele was observed (Figure 2).
- At the time of surgical planning, the infection had shown partial clinical stabilization, evidenced by reduction in purulent discharge, decreased conjunctival congestion, absence of progression of corneal infiltrates, and clinical response to intensive systemic and topical antimicrobial therapy following microbiological confirmation. However, despite medical treatment, persistent peripheral corneal thinning with impending risk of perforation remained. In view of this tectonic threat, early surgical intervention was undertaken as a globe‐preserving and anti‐inflammatory measure rather than for optical rehabilitation.
- Bilateral amniotic membrane grafting with perilimbal conjunctival excision and bandage contact lens placement was performed.
- For syphilis, benzathine penicillin G 2.4 million units intramuscularly weekly for 3 weeks was administered.

## 4 Results (Outcome and Follow‐Up)

- Follow‐up at 6 weeks: visual acuity 6/9 bilaterally, healed ulcers, clear corneas, no recurrence.

## 5 Discussion

Adult gonococcal conjunctivitis is an uncommon but aggressive ocular infection. The organism's ability to penetrate intact epithelium explains its rapid progression to keratitis and corneal ulceration [1, 3]. If untreated, complications may include perforation, endophthalmitis, and blindness [2].

Our case is unusual due to the development of bilateral peripheral ulcerative keratitis, a rare manifestation. Similar cases are sparsely reported in literature [4, 5]. This highlights the destructive potential of ocular gonorrhea, even in immunocompetent adults.

Management of ocular gonorrhea requires systemic antibiotics. Current CDC guidelines recommend intramuscular ceftriaxone 500 mg (1 g if > 150 kg) plus azithromycin to cover possible *Chlamydia trachomatis* [6]. In this case, intravenous ceftriaxone 1 g twice daily was chosen due to severe corneal involvement and rapid progression, while doxycycline was added both for possible chlamydial co‐infection and to reduce collagenolysis. The absence of culture/NAAT and chlamydia testing is a limitation.

Topical fluoroquinolones (moxifloxacin) were initially given empirically for broad coverage but discontinued once gonococcal infection was established, acknowledging high resistance rates.

Surgical management with amniotic membrane grafting proved crucial in preventing corneal perforation. This technique provides structural support, reduces inflammation, and promotes epithelial healing.

The concurrent diagnosis of late latent syphilis underscores the importance of comprehensive STI screening in patients with gonococcal infection. Benzathine penicillin G remains the standard of care.

## 6 Conclusion

This case demonstrates that adult gonococcal conjunctivitis can progress rapidly to peripheral ulcerative keratitis. Early microbiological evaluation, broad STI screening, systemic ceftriaxone therapy, and timely surgical intervention with amniotic membrane grafting are key to preserving vision.

## Author Contributions

**Prashim Sharma:** conceptualization, data curation, methodology, visualization, writing – original draft. **Ramesh Bikram K.C.:** formal analysis, validation. **Alisha Adhikari:** project administration, resources, writing – review and editing. **Badri Prasad Badhu:** writing – review and editing. **Prerna Arjyal Kafle:** investigation, writing – review and editing.

## Funding

The authors have nothing to report.

## Consent

Written informed consent was obtained from the patient for publication of this case report and any accompanying images.

## Data Availability

Data available on request from the authors.

## References

[1] J. S. Lee , H. Y. Choi , J. E. Lee , S. H. Lee , and B. S. Oum , “Gonococcal Keratoconjunctivitis in Adults,” Eye (London, England) 16, no. 5 (2002): 646–649. 12194086 10.1038/sj.eye.6700112

[2] M. V. Humbert and M. Christodoulides , “Atypical, Yet Not Infrequent, Infections With Neisseria Species,” Pathogens 9, no. 1 (2019): 10. 31861867 10.3390/pathogens9010010 PMC7168603

[3] J. Costumbrado , D. K. Ng , and S. Ghassemzadeh , “Gonococcal conjunctivitis,” in StatPearls (StatPearls Publishing, 2023). 29083770

[4] S. Ullman , T. J. Roussel , and R. K. Forster , “Gonococcal Keratoconjunctivitis,” Survey of Ophthalmology 32, no. 3 (1987): 199–208. 2965423 10.1016/0039-6257(87)90095-6

[5] L. McAnena , S. J. Knowles , A. Curry , and L. Cassidy , “Prevalence of Gonococcal Conjunctivitis in Adults and Neonates,” Eye (London, England) 29, no. 7 (2015): 875–880. 25907207 10.1038/eye.2015.57 PMC4506339

[6] K. A. Workowski , L. H. Bachmann , P. A. Chan , et al., “Sexually Transmitted Infections Treatment Guidelines, 2021,” MMWR – Recommendations and Reports 70, no. 4 (2021): 1–187. 10.15585/mmwr.rr7004a1 PMC8344968 34292926

